# In Vivo Evaluation of Gamma-Irradiated and Heparin-Immobilized Small-Diameter Polycaprolactone Vascular Grafts with VEGF in Aged Rats

**DOI:** 10.3390/polym14061265

**Published:** 2022-03-21

**Authors:** Se-Eun Kim, Sung-In Jeong, Kyung-Mi Shim, Kwangsik Jang, Jong-Seok Park, Youn-Mook Lim, Seong-Soo Kang

**Affiliations:** 1 BK21 FOUR Program, Department of Veterinary Surgery, College of Veterinary Medicine, Chonnam National University, Gwangju 61186, Korea; ksevet@jnu.ac.kr (S.-E.K.); simchung-98@hanmail.net (K.-M.S.); rhkdtlr0327@nate.com (K.J.); 2 Biomaterial R&BD Center, Chonnam National University, Gwangju 61186, Korea; 3 Advanced Radiation Technology, Korea Atomic Energy Research Institute, Jeongeup-si 56212, Korea; sijeong@kaeri.re.kr (S.-I.J.); jspark75@kaeri.re.kr (J.-S.P.)

**Keywords:** small-diameter vascular graft, polycaprolactone, gamma irradiation, heparin, VEGF, aged rat

## Abstract

The effectiveness of small-diameter vascular grafts depends on their antithrombogenic properties and ability to undergo accelerated endothelialization. The extreme hydrophobic nature of poly(ε-caprolactone) (PCL) hinders vascular tissue integration, limiting its use in medical implants. To enhance the antithrombogenicity of PCL as a biomaterial, we grafted 2-aminoethyl methacrylate (AEMA) hydrochloride onto the PCL surface using gamma irradiation; developed a biodegradable heparin-immobilized PCL nanofibrous scaffold using gamma irradiation and *N*-(3-dimethylaminopropyl)-*N*′-ethyl carbodiimide hydrochloride/*N*-hydroxysuccinimide reaction chemistry; and incorporated vascular endothelial growth factor (VEGF) into the scaffold to promote vascular endothelial cell proliferation and prevent thrombosis on the vascular grafts. We assessed the physicochemical properties of PCL, heparin-AEMA-PCL (H-PCL), and VEGF-loaded heparin-AEMA-PCL (VH-PCL) vascular grafts using scanning electron microscopy, attenuated total reflection–Fourier transform infrared spectroscopy, toluidine blue O staining, and fibrinogen adsorption and surface wettability measurement. In addition, we implanted the vascular grafts into 24-month-old Sprague Dawley rats and evaluated them for 3 months. The H-PCL and VH-PCL vascular grafts improved the recovery of blood vessel function by promoting the proliferation of endothelial cells and preventing thrombosis in clinical and histological evaluation, indicating their potential to serve as functional vascular grafts in vascular tissue engineering.

## 1. Introduction

Blood vessels, present throughout the body, deliver nutrients, oxygen, and other active substances to the tissues, as well as clearing them of cellular by-products and carbon dioxide, thereby playing a crucial role in the maintenance of physiological homeostasis [1,2]. However, vascular aging can cause morphological and functional changes that lead to luminal narrowing, decreased flow, and distal tissue ischemia. The incidence of cardiovascular diseases, such as stroke and heart disease, is increasing with the rise in the global aging population. Since most vascular diseases are treated by vascular bypass using vascular grafts [1,3], the demand for blood vessel transplants is also increasing [4].

Autografts (from self) and homografts (from others) are used in vascular bypass surgeries. However, autografts need a prolonged operative time, increase the risk of peri-operative infection, and allow for limited harvested graft length [5,6,7]. Meanwhile, homografts degenerate quickly in the arterial circulation and can be used only selectively [8]. To overcome these disadvantages, synthetic vascular grafts have been developed. Conventional synthetic vascular grafts, such as those made of polyethylene terephthalate (Dacron) and expanded polytetrafluoroethylene (ePTFE), are effective for the surgical treatment of large- and medium-sized vessels [9]. However, their use as small-diameter vascular grafts is limited because of high rates of early occlusion, secondary to post-grafting thrombosis, and they are also prone to intimal hyperplasia [10,11].

Small-diameter vascular grafts can be processed using several methods such as extrusion and expansion, electrospinning, thermally induced phase separation, braiding, 3D printing, hydrogel tubing, and gas foaming [12]. Electrospinning is an efficient method of producing nanofibrous structures for natural and synthetic polymers. The large pore size, thickness, and a tunable surface-area-to-volume ratio are important features of electrospun scaffolds that enhance cellular adhesion, proliferation, and infiltration [13,14].

Many synthetic polymers, such as polycaprolactone (PCL), poly(lactide-*co*-glycolide) (PLGA), and polylactic acid (PLA), have been successfully electrospun into nanofibers with excellent mechanical properties and biocompatibility. However, despite their biocompatibility and biodegradability, synthetic scaffolds have generally poor cell affinity due to their low hydrophilic properties and a lack of cell recognition sites. Therefore, small-diameter synthetic vascular grafts tend to exhibit unsatisfactory long-term results [15,16].

In a previous study, we grafted electrospun PCL nanofibers with amino groups of 2-aminoethyl methacrylate (AEMA) using gamma irradiation [17]. Gamma irradiation induces graft polymerization, where the associated energy uniformly free-radicalizes the reaction between acrylate monomers and polymers [18,19,20]. In this study, to overcome the disadvantages of small-diameter vascular grafts, we developed synthetic vascular grafts with electrospun, tubular, surface-modified PCL nanofibers. Using gamma irradiation, we added heparin and vascular endothelial growth factor (VEGF) onto the vascular grafts. The carboxyl groups of heparin were immobilized using the *N*-(3-dimethylaminopropyl)-*N*′-ethyl carbodiimide hydrochloride (EDC)/*N*-hydroxysuccinimide (NHS) reaction to sustain VEGF [17]. Both VEGF and heparin are essential for the endothelialization of vascular graft materials, and act as signaling proteins that affect the metabolism of endothelial cells and prevent thrombogenesis [21,22]. VEGF helps in vessel regeneration by stimulating the proliferation and migration of endothelial cells and promoting a complex series of re-endothelialization.

Many in vivo evaluations of artificial vascular grafts have been performed on young and adult rats with body weights of 200–500 g [23,24,25,26,27]. According to Pallav, male rats weighing 500 g are younger than ~8–10 months old [28]. In addition, rats aged 24 months show decreased vascular development or cardiovascular function with aging [29,30,31]. In this study, we used Sprague Dawley (SD) rats aged 24 months and weighing 600–800 g to recreate physiological and vascular conditions similar to those in older people aged ~60 years. To date, no studies have been conducted in aged rats. This is the first study to use aged rats to simulate the vascular environment of humans at the age at which they would be most likely to require a transplant. In addition, arterial wall aging in rats is similar to that in humans, including endothelial dysfunction, diffuse intimal thickening, increased wall stiffness, and decreased nitric oxide bioavailability. Therefore, experiments can be performed in a similar environment in older people [32,33].

In this study, we manufactured VEGF-loaded heparin-AEMA-PCL (VH-PCL) vascular grafts, implanted them into 24-month-old SD rats, and evaluated it for patency. The histological analysis evaluated the biocompatibility and efficiency of the grafted vascular scaffolds. The findings will help demonstrate the feasibility of this graft modification method for vascular tissue engineering.

## 2. Materials and Methods

### 2.1. Materials

We obtained PCL (440744) of molecular weight 80,000 g/mol, AEMA (900652), TBO (198161), fluorescamine (F9015), EDC (03449), and 2-(*N*-morpholino) ethane sulfonic acid sodium salt (MES) (M3671) from Sigma-Aldrich (St. Louis, MO, USA). Tetrahydrofuran (THF) (0214) and *N*, *N*-dimethylformamide (DMF) (1369) were purchased from Duksan Reagents and Chemicals (Ansan, Korea). All other reagents and solvents were of analytical grade and were used as received.

### 2.2. Preparation of Heparin-Immobilized and VEGF-Loaded Methacrylated PCL Vascular Grafts

PCL was dissolved in a mixture of THF and DMF (70:30 *v*/*v*) at 21 °C to a final concentration of 13% (*w*/*v*). The solution was loaded into a 12 mL plastic syringe (Henke Sass Wolf, Tuttlingen, Germany) through a blunt 21 G stainless steel needle (NanoNC, Seoul, Korea). A custom-designed electrospinning process was constructed using an infusion pump (SHB366; Sckjmotor, Shenzhen, China), and the needle was connected to a high-voltage power supply (ESR-200RD; NanoNC, Seoul, Korea). The solution flow-rate, applied voltage, and spinning time were set at 1 mL/h, 11.3 kV, and 1 h, respectively, while the distance between the stainless-steel blunt-ended needle tip (outer diameter of 0.81 mm, inner diameter of 0.51 mm) and the custom-made rotating stainless steel collector (outer diameter of 1.3 mm, length of 25 cm; Four Leaves Inc., Osaka, Japan) was fixed at 15 cm. The small-diameter, tubular electrospun PCL vascular grafts were dried overnight at 21 °C. Next, the vascular grafts were placed in AEMA/methanol solution (AEMA concentration 7% *w*/*v*) in a 30 mL glass vial, and AEMA was grafted onto the vascular grafts using 60 Co gamma irradiation at 25 kGy and a dose rate of 10 kGy/h at 21 °C (MDS Nordion, Ottawa, ON, Canada, IR 221 n wet storage type C-188 at the Korea Atomic Energy Research Institute, Jeongeup-si, Korea) [17]. The samples were washed with distilled water (DW) for 48 h at 21 °C to remove unreacted monomers and homopolymers. To immobilize heparin onto the AEMA-PCL nanofibers (H-PCL), the samples were immersed in 0.1 M MES buffer solution (pH 5) for 30 min. Next, they were immersed overnight at 21 °C in 5 mg/mL of EDC/NHS and 2 mg/mL of heparin dissolved in 0.1 M MES buffer solution. The samples were washed with DW for 48 h to remove unreacted monomers and homopolymers. The electrospun H-AEMA-PCL grafts were cut into a round shape (inner diameter: 1.1 mm; length: 10 mm) and placed in 2 mL Eppendorf (EP) tubes. Then, a solution of VEGF in 100 ng/mL of phosphate-buffered saline (PBS) was added to each well and incubated at 37 °C for 1 h to load VEGF onto the H-AEMA-PCL grafts (VH-PCL) [22].

### 2.3. Characterization of Nanofibers

#### 2.3.1. Scanning Electron Microscopy

Surface and cross-sectional morphologies of unmodified PCL, H-PCL, and VH-PCL vascular grafts were sputter-coated with a layer of gold for 60 s and visualized using SEM (JSM-6390; JEOL, Akishima, Japan) at an operating voltage of 5 kV.

#### 2.3.2. Attenuated Total Reflection–Fourier Transform Infrared Spectroscopy

To characterize surface properties, the infrared spectra of PCL, H-PCL, and VH-PCL vascular grafts were analyzed using ATR-FTIR (Bruker TENSOR 37; Bruker AXS. Inc., Karlsruhe, Germany). The transmission and ATR-FTIR spectra between 4000 and 600 cm^−1^ were measured at a 4 cm^−1^ resolution, averaging 64 scans/sample.

#### 2.3.3. Mechanical Properties

The tensile properties of PCL, H-PCL, and VH-PCL vascular grafts were measured using a DB35-10 Universal Testing Machine (Bongshin Loadcell Co., Ltd., Osan, Korea). A 5 N maximum load cell with a cross-head speed of 1 mm/min was used. Burst pressure testing was performed using a catheter balloon pressure syringe (Genoss Co., Ltd., Suwon, Korea). Briefly, a 20 mL pressure syringe was filled with Dulbecco’s Modified Eagle’s Medium, which was inserted into a 20 mm section of each graft that was connected directly to the catheter balloon. The pressure was increased to 760 mmHg/min via a high-pressure gage (pressure range: 0–200 PSI; Genoss Co., Ltd., Suwon, Korea) before rupture. The burst pressure was recorded as the maximum pressure before rupture.

#### 2.3.4. Toluidine Blue O Assay

To confirm the grafting of heparin, TBO staining and quantification were investigated. TBO is reacted with carboxylic acid, so it is used to stain and quantify heparin. PCL, H-PCL, and VH-PCL vascular grafts were stained and immersed in TBO solution (0.1 M HCl, 60 mg NaCl, and 12 mg TBO chloride) for 4 h at 21 °C. The TBO-stained samples were resolved for 12 h at 21 °C in a mixture of 0.1 M NaOH and ethanol (1:4 *v*/*v*) until complete decolorization. The amount of TBO bound to the carboxylic acid group of heparin on AEMA-PCL vascular grafts was quantified by measuring the absorbance of each well at 630 nm using a Powerwave XS microplate reader (Bio Tek Instruments Inc., Winooski, VT, USA).

#### 2.3.5. Water Contact Angle

Many studies have proved that enhancing the surface wettability of scaffolds leads to increased cell adhesion and spreading. The wettability of the electrospun PCL and H-PCL nanofiber sheets (3 mm diameter) was analyzed by measuring the water contact angle on dry samples using a Phoenix-300 contact angle meter (Surface Electro Optics Co., Ltd., Suwon, Korea). The droplet size was set to 10 μL. Five samples from each graft were used for each test.

#### 2.3.6. Fibrinogen Adsorption

The electrospun PCL and H-PCL nanofiber sheets (3 mm diameter) were immersed in 1 mL ethanol for 1 min and washed thrice with 5 mL DW for 5 min. Before adsorption, the pre-wetted samples were placed in a 200 μL fibrinogen solution (0.6 μg/mL in PBS) and incubated at 37 °C for 2 h under static conditions. Then, the samples were washed five times with PBS for 15 min to remove the unbound fibrinogen. After washing, the samples were immersed in 200 μL of 1 wt% sodium dodecyl sulfate (SDS) solution with gentle shaking for 1 h, to remove the adsorbed fibrinogen from the surface of the sample at room temperature. The amount of adsorbed fibrinogen was determined by measuring the amount of fibrinogen in the SDS solution using a micro–bicinchoninic acid (BCA) protein assay kit (Thermo Fisher Scientific, Waltham, MA, USA) and a Cytation plate reader (Bio Tek Instrument Inc., Winooski, VT, USA).

### 2.4. Implantation of Vascular Grafts into Aged Rats

#### 2.4.1. Animals

We used 15 male SD rats aged 24 months and weighing 600–800 g (Samtaco Bio Co., Osan, Korea) in this study. The rats were housed in an air-conditioned room with a 12 h/12 h light/dark cycle under controlled temperature (23 °C ± 2 °C), illumination intensity (200–300 Lux), and humidity (55% ± 10%). Drinking water and commercial rodent diet (Samyang Feed, Daejeon, Korea) were provided ad libitum.

The study protocols were approved by the Institutional Animal Care and Use Committee of Chonnam National University, Korea (CNU IACUC-YB-2017-25), and the animals were cared for according to the Guidelines for Animal Experiments of Chonnam National University, Korea.

#### 2.4.2. Vascular Graft Implantation

PCL, H-PCL, and VH-PCL vascular grafts were implanted using the standard microsurgical technique. Briefly, SD rats were anesthetized with an intraperitoneal injection of 10 mg/kg of xylazine hydrochloride (Rompun^®^; Bayer Korea, Seoul, Korea) and 20 mg/kg of tiletamine-zolazepam (Zoletil 50^®^; Virbac, Seoul, Korea). A midline laparotomy incision was made, and the infrarenal abdominal aorta was isolated by blunt and sharp dissection. After clamping proximally below the renal arteries and distally above the aorto-iliac bifurcation, a 1 cm segment of the abdominal aorta was resected. The PCL, H-PCL, and VH-PCL vascular grafts (inner diameter: 1.1 mm; length: 1 cm) were implanted. All grafts were harvested 3 months post-implantation for analysis.

### 2.5. In Vivo Evaluation of Vascular Grafts in Aged Rats

#### 2.5.1. Ultrasonography

Ultrasonography was used to monitor the PCL, H-PCL, and VH-PCL vascular grafts at 4 and 12 weeks post-implantation. The luminal diameter of the vascular grafts was determined using sonography, and patency was determined by measuring the flow velocity both proximal and distal to the vascular grafts. In addition, a color ultrasound with a 10 MHz linear transducer was used to monitor acute thrombosis and hemodynamic changes.

#### 2.5.2. Angiography

Angiography was performed at 12 weeks post-implantation. Briefly, the rats were anesthetized, as described above, and angiography was performed in vivo with a KMC-950 fluoroscope (Komed Medical System, Kwangju, Korea) using a contrast agent injection (Omnipaque^®^, 3 mL/total) through a carotid artery catheter.

#### 2.5.3. Scanning Electron Microscopy for Harvested Vascular Grafts

The rats were sacrificed, and the vascular grafts were harvested. The harvested grafts were fixed in 2.5% (*v*/*v*) glutaraldehyde for 30 min, dehydrated using a graded ethanol series, and dried. The intimal surfaces of the dried specimens were sputter-coated with gold for SEM imaging.

#### 2.5.4. Histological Evaluation of Harvested Vascular Grafts

The harvested grafts were rinsed with saline and immersed in 10% neutral buffered formalin. Then, the vascular grafts were sliced into 4-mm-thick longitudinal and transverse sections and stained with hematoxylin and eosin (H&E) and Masson’s trichrome (MT). The blood vessel thickness data were collected by averaging each animal based on 4 positions (12 o’clock, 3 o’clock, 6 o’clock, and 9 o’clock) in the circular blood vessel shape in the H&E staining slide, and statistical analysis was performed by collecting the data again within each group.

#### 2.5.5. Immunofluorescence Staining

The materials for immunohistochemical staining used in this study were 4′,6-diamidino-2-phenylindole (DAPI) mounting solution from Vector Laboratories Inc. (Burlingame, CA, USA), fluorescein isothiocyanate (FITC)-conjugated streptavidin from Cell Signaling Technology^®^ (Danvers, MA, USA), anti-cluster of differentiation 31 (CD31) antibody from Thermo Fisher Scientific (Cheshire, UK), and alpha–smooth muscle actin (α-SMA) antibody from Abcam (Cambridge, UK). Bovine serum albumin (BSA) was obtained from AMRESCO^®^ (Solon, OH, USA) and goat serum from Gibco Life Technologies^TM^ (Carlsbad, CA, USA). The other chemical reagents used were purchased from Sigma-Aldrich.

After complete drying, the vascular graft sections were deparaffinized with xylene, hydrated with ethanol, and washed with TBS-T buffer (0.05 mM Trizma^®^ base, 0.05% Triton^®^-X-100) (pH 7.6). Next, the sections were dipped in 10 mM citrate buffer (pH 6) at 90 °C for 30 min, treated with blocking buffer (BB; TBS-T buffer with 2% BSA solution, 10% goat serum) at 37 °C for 1 h, and then incubated overnight with a primary antibody, anti-CD31 (1:40 diluted in BB) or anti-SMA (1:100 diluted in BB), at 4 °C. Subsequently, they were treated with a secondary antibody, biotin-conjugated goat anti-mouse immunoglobulin G (IgG) (1:200 diluted in BB), at 37 °C for 1 h, followed by treatment with streptavidin-FITC (1:200 diluted in BB) at 37 °C for 1 h. Finally, the sections were mounted with VECTASHIELD^®^ Mounting Medium with DAPI for nuclei staining. The images of fluorescence-stained tissues were captured using a fluorescence microscope.

#### 2.5.6. Statistical Evaluation

All quantitative results were obtained from more than three samples. The comparison between two groups was analyzed using the paired Student’s *t*-test and expressed as a mean ± standard deviation (SD). When comparing three or more groups, the analysis was conducted using the Kruskal–Wallis test within the entire group and then, post-hoc, was conducted by the Dunn test between each group. These data were expressed as medians. All analysis of the data was conducted using SPSS Statistics version 27.0 (SPSS Inc., Chicago, IL, USA) and GraphPad Prism version 5.0 (Graphpad Software Inc., San Diego, CA, USA), and a value of *p* < 0.05 was considered to be statistically significant.

## 3. Results

We developed electrospun tubular PCL vascular scaffolds with AEMA grafting using a gamma-irradiation technique. AEMA grafting was used to prepare the electrospun PCL for the subsequent covalent immobilization of heparin using the EDC/NHS reaction.

**Figure 1:**
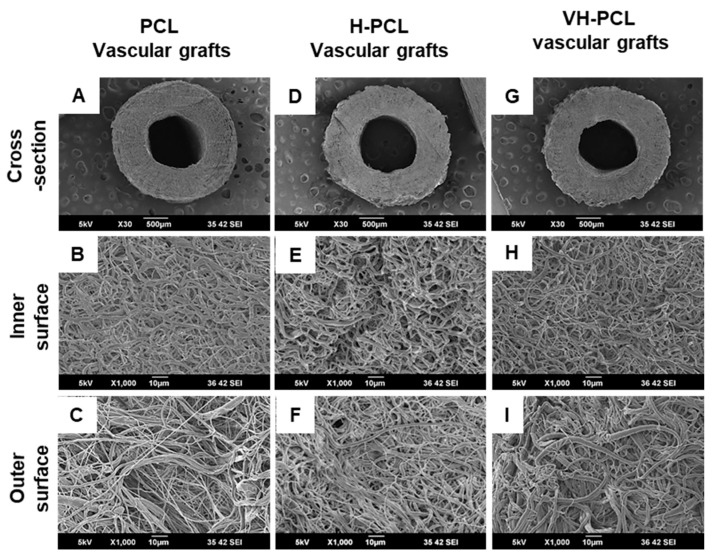
Morphologies of unmodified PCL, H-PCL, and VH-PCL vascular grafts: (**A**–**I**) SEM micrographs. PCL—poly(ε-caprolactone); H-PCL—heparin-AEMA-PCL; AEMA—2-aminoethyl methacrylate; VH-PCL—vascular endothelial growth factor (VEGF)-loaded heparin-AEMA-PCL; SEM—scanning electron microscopy.

SEM images revealed that the histoarchitecture of the tubular electrospun vascular grafts was roughly spherical in shape, like that of the native aorta (Figure 1). The cross section of the PCL, H-PCL, and VH-PCL vascular grafts showed a highly porous, compact, and homogeneous fibrous structure similar to that of the native aorta (Figure 1A–I). The average inner diameter, wall thickness, and fiber diameter of the electrospun vascular grafts were approximately 1.5 mm, 600–650 mm, and 1.3 mm, respectively. There average inner diameter, wall thickness, and fiber diameter of the PCL and H-PCL vascular grafts were slightly smaller after gamma irradiation. However, there was difference in these three parameters before and after VEGF loading in the H-PCL vascular grafts.

**Figure 2:**
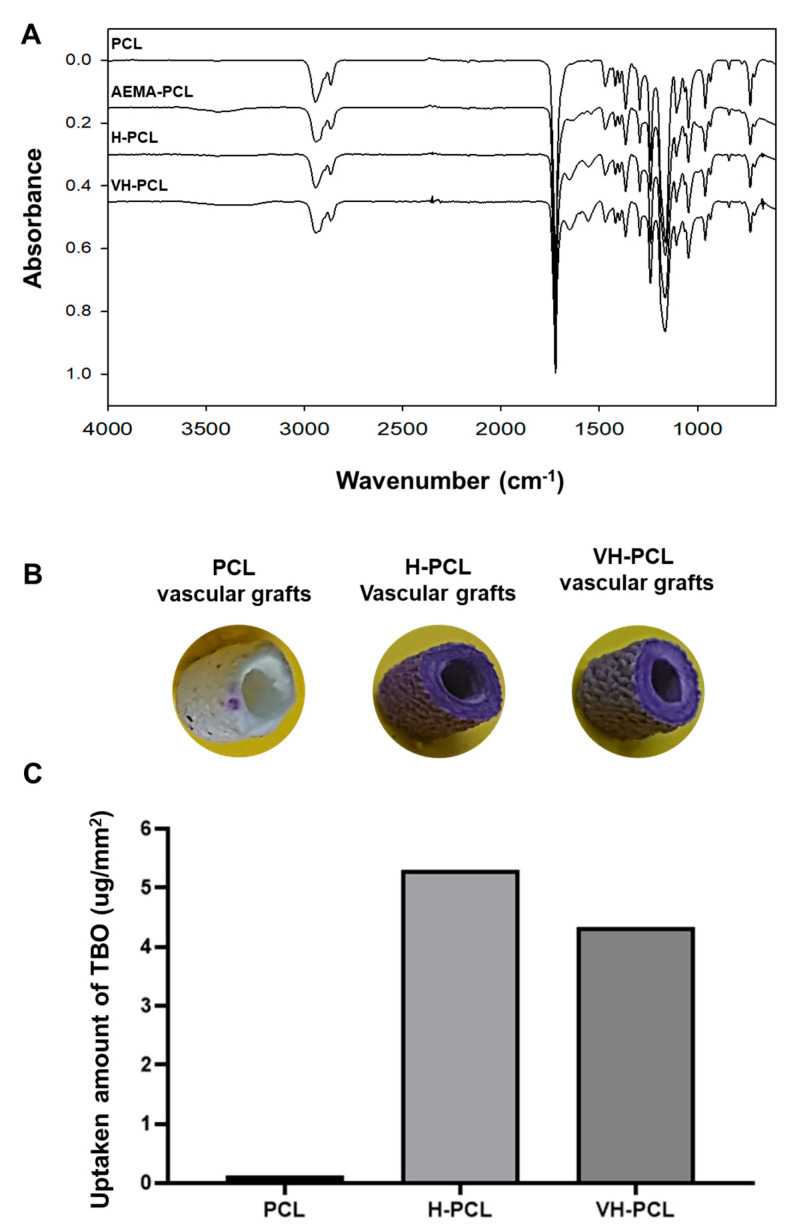
Surface characterization of unmodified PCL, H-PCL, and VH-PCL vascular grafts: (**A**) ATR-FTIR spectrum; (**B**) TBO staining; and (**C**) heparin surface density (the graph values shown are medians). PCL (*n* = 3)—poly(ε-caprolactone); H-PCL (*n* = 3)—heparin-AEMA-PCL; AEMA—2-aminoethyl methacrylate; VH-PCL (*n* = 3)—vascular endothelial growth factor (VEGF)-loaded heparin-AEMA-PCL; ATR-FTIR—attenuated total reflection–Fourier transform infrared spectroscopy; TBO—toluidine blue O.

Covalent modifications of the PCL, H-PCL, and VH-PCL vascular grafts were assessed using ATR-FTIR analysis (Figure 2A). The characteristic peaks for amino and amine groups of AEMA-PCL were observed at 1610 and 3300 cm^−1^, respectively. The distinctive peaks for the amide group of AEMA and a carboxylic acid group of heparin were detected at 1650 and 1550 cm^−1^, respectively, in the H-PCL vascular grafts, before and after VEGF loading. In addition, these signals disappeared after heparin conjugation, indicating that the amines formed amide bonds with the carboxyl groups of heparin.

The surface densities of the PCL, H-PCL, and VH-PCL vascular grafts were quantified using a TBO staining assay (Figure 2B,C). Although the PCL vascular grafts did not change color, heparin on the surfaces of the H-PCL and VH-PCL vascular grafts were stained purple because the anionic ions of the carboxylic acid groups of heparin were bound to the cationic TBO dye (Figure 2B). The heparin surface densities of the PCL, H-PCL, and VH-PCL vascular grafts were approximately 0.29 ± 0.39, 5.16 ± 0.86, and 4.67 ± 0.70 mg/mm^2^, respectively (Figure 2C).

**Figure 3:**
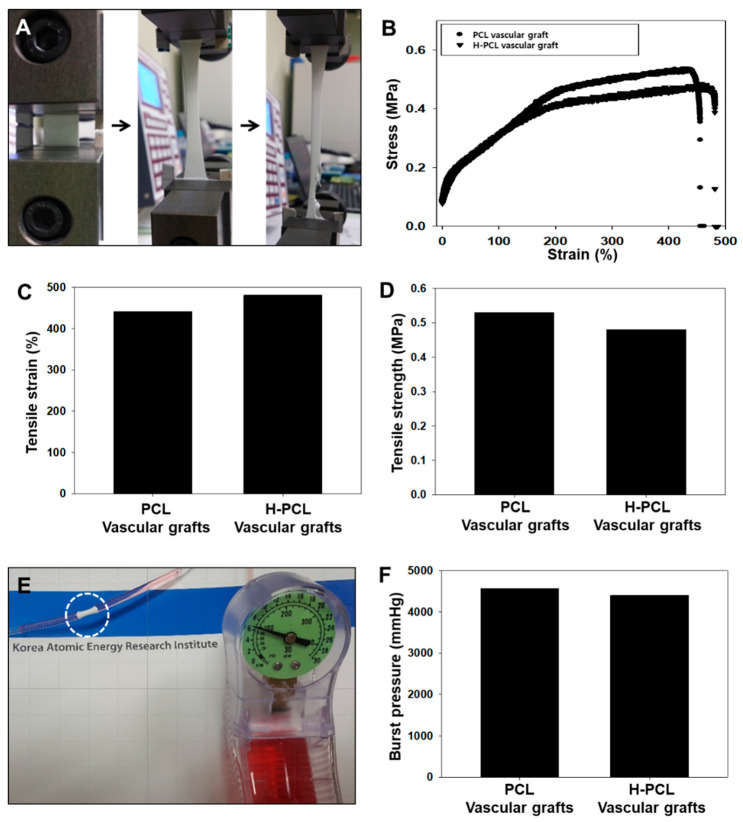
Mechanical properties of unmodified PCL and H-PCL vascular grafts: (**A**) Optical image of elongation at break; (**B**) representative tensile strain–stress curves; (**C**) tensile strain (*n* = 1); (**D**) tensile strength (*n* = 1); (**E**) visualization of burst pressure testing; and (**F**) burst pressure (*n* = 1). PCL—poly(ε-caprolactone); H-PCL—heparin-AEMA-PCL; AEMA—2-aminoethyl methacrylate.

Constant strain rate and burst pressure tests were performed to determine the specific elongation at break, tensile strength, and burst pressure properties of the PCL and H-PCL vascular grafts (Figure 3). A typical representative stress–strain curve is shown in Figure 3A,B. There was no significant difference in the ultimate strain at break for the PCL and H-PCL vascular grafts (Figure 3C). The elongation at break for the PCL vascular grafts (442%) was significantly lower than that for the H-PCL vascular grafts (481%). As shown in Figure 3D–F, the tensile strength and burst pressure of the PCL vascular grafts (0.52 MPa and 4560 mmHg, respectively) were higher than those of the H-PCL vascular grafts (0.47 MPa and 4400 mmHg, respectively), indicating that heparin immobilization helps reinforce electrospun vascular grafts.

**Figure 4:**
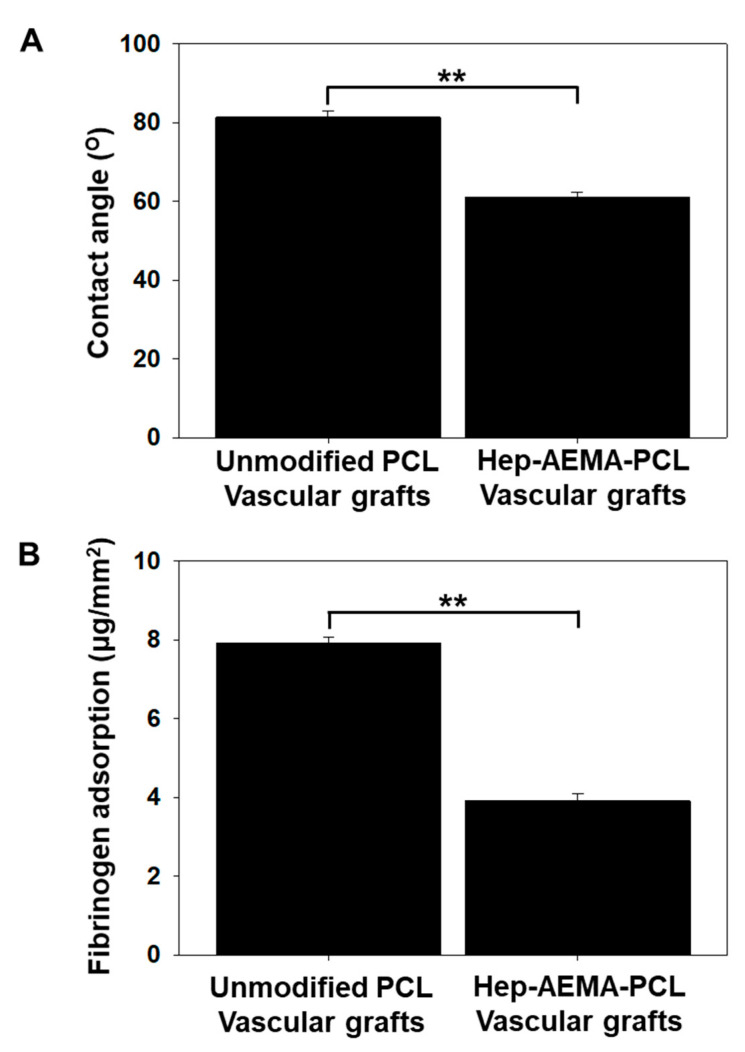
Water contact angle (**A**) and fibrinogen adsorption (**B**) of unmodified PCL and H-PCL vascular grafts (the graph values shown are means ± standard deviation; ** Significance in *p* < 0.01 relative to unmodified PCL). PCL (*n* = 3)—poly(ε-caprolactone); H-PCL (*n* = 3)—heparin-AEMA-PCL; AEMA—2-aminoethyl methacrylate.

Water contact angle measurements were performed to analyze the wettability behavior of the PCL and H-PCL vascular grafts (Figure 4A). The water contact angle for the PCL vascular grafts reached 83° due to the hydrophobic nature of PCL. After modification with heparin and AEMA grafting, the water contact angle of the H-PCL vascular grafts decreased to 61°.

The micro-BCA assay (Figure 4B) showed a significantly lower amount of negatively charged fibrinogens adhered to the anionic surface of the H-PCL vascular grafts (3.91 ± 0.17 mg/mm^2^) than of the PCL vascular grafts (7.92 ± 0.12 mg/mm^2^). The anionic surface of the H-PCL vascular grafts could inhibit the adsorption of negatively charged fibrinogens via electrostatic charge repulsion, indicating better blood compatibility than the PCL vascular grafts.

**Figure 5:**
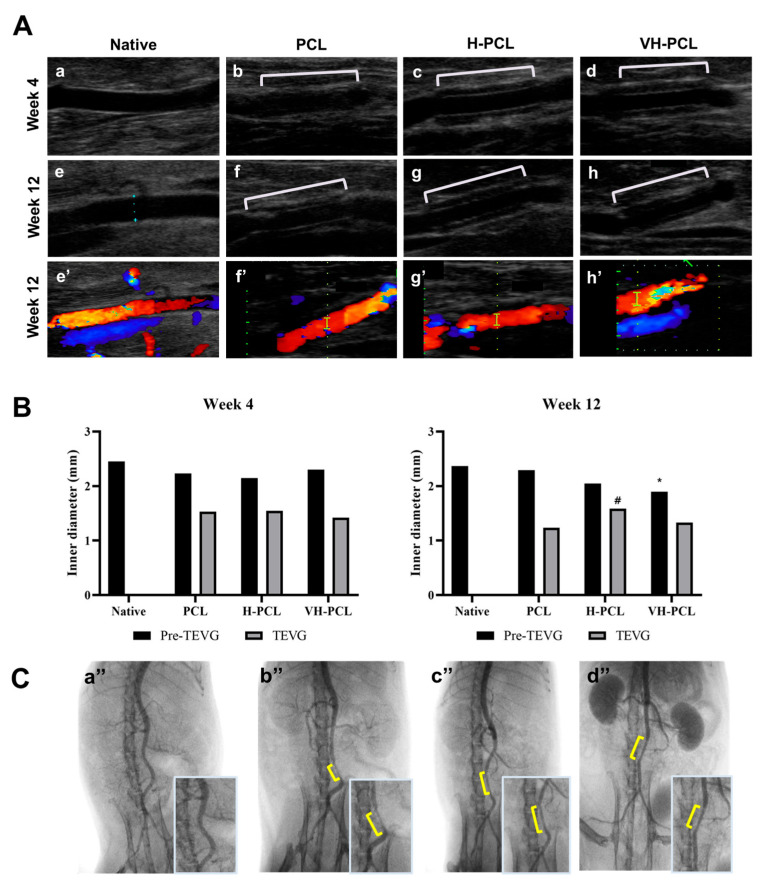
(**A**) Doppler ultrasound examination of the normal rat (**a**,**e**), unmodified PCL (**b**,**f**), H-PCL (**c**,**g**), and VH-PCL vascular grafts (**d**,**h**) at 4 and 12 weeks post-implantation. Color doppler ultrasound examination of the normal rat (**e’**), unmodified PCL (**f’**), H-PCL (**g’**), and VH-PCL vascular grafts (**h’**) at 12 weeks post-implantation; (**B**) inner diameter measurement using ultrasound at 4 and 12 weeks post-implantation (the graph values shown are medians; *: Significance in *p* < 0.05 relative to Native; ^#^: Significance in *p* < 0.05 relative to PCL); (**C**) representative angiographic images at 12 weeks post-implantation: (**a’’**) normal rat, (**b’’**) unmodified PCL, (**c’’**) H-PCL, and (**d’’**) VH-PCL groups. Native (*n* = 3)—normal rat; PCL (*n* = 4)—poly(ε-caprolactone); H-PCL (*n* = 4)—heparin-AEMA-PCL; AEMA—2-aminoethyl methacrylate; VH-PCL (*n* = 4)—vascular endothelial growth factor (VEGF)-loaded heparin-AEMA-PCL; Pre-TEVG—front portion of the implanted graft; TEVG—tissue-engineered vascular graft.

During this in vivo study, no bleeding was observed from any of the vascular grafts post-implantation. The inner diameter and patency of the implanted vascular grafts at 4 and 12 weeks post-implantation were evaluated using ultrasonography (Figure 5). The inner diameter was also measured on the frontal site of the vascular grafts (pre-graft) and the vascular graft site (graft). All the vascular grafts showed patency at 4 and 12 weeks post-implantation, while the internal diameter of the PCL vascular grafts increased to 1.53 mm at 4 weeks, and then decreased to 1.24 mm at 12 weeks post-implantation. Although the H-PCL and VH-PCL vascular grafts also showed increased inner diameters of 1.54 and 1.42 mm, respectively, at 4 weeks post-implantation, they displayed nearly similar diameters at 12 weeks post-implantation.

Arterial angiography performed at 12 weeks post-implantation to evaluate the patency of the implanted PCL, H-PCL, and VH-PCL vascular grafts showed patency with a normal vessel-like appearance and without graft rupture, dilatation, or aneurysm. However, the PCL vascular grafts had a narrower inner diameter than the H-PCL and VH-PCL vascular grafts did (Figure 5).

**Figure 6:**
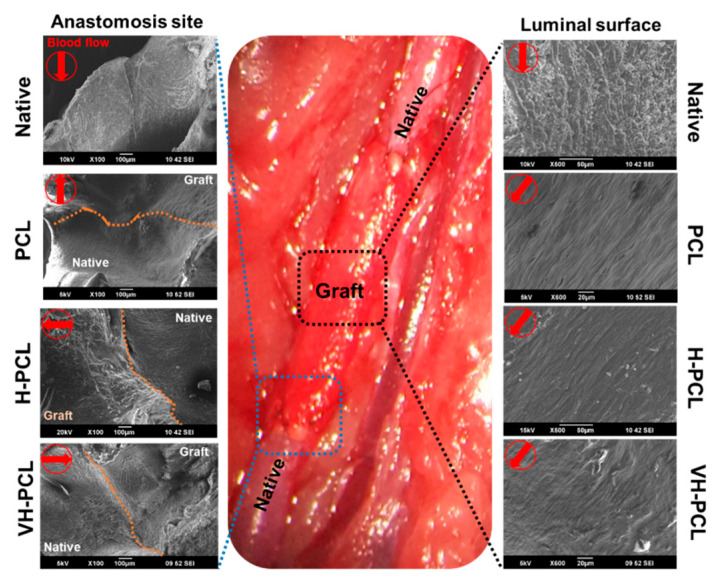
Representative SEM images of vascular anastomosis site at 12 weeks post-implantation. All of the SEM images at anastomosis site were observed at a magnification of ×100. On luminal surface, Native was observed at ×500 and the rest at ×600. Native—normal vessel in the rat; SEM—scanning electron microscopy; PCL—poly(ε-caprolactone); H-PCL—heparin-AEMA-PCL; AEMA—2-aminoethyl methacrylate; VH-PCL—vascular endothelial growth factor (VEGF)-loaded heparin-AEMA-PCL.

After ultrasonography and angiography, the implanted vascular grafts were harvested to evaluate their biocompatibility using SEM and histological methods. SEM analysis showed patency with no strictures in all PCL, H-PCL, and VH-PCL vascular grafts. The luminal surface of the PCL, H-PCL, and VH-PCL vascular grafts showed appropriate integration with the native aorta and endothelialization at 12 weeks post-grafting (Figure 6). The endothelial cells showed alignment along with the bloodstream. Among all the experimental groups, the luminal surface of the VH-PCL vascular grafts was the closest in appearance to native vessels, while that of the PCL vascular grafts was the least similar.

**Figure 7:**
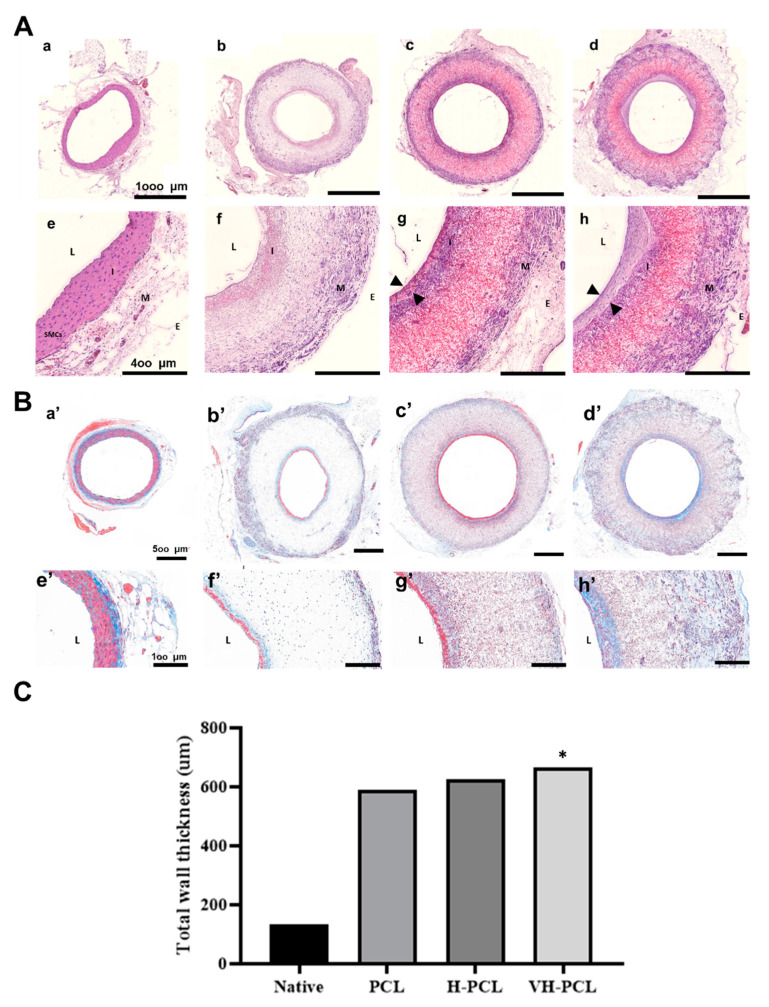
Histological images and measurement of vascular wall thickness in TEVGs (*n* = 4) and normal (*n* = 3) rats at 12 weeks post-implantation: (**A**) H&E staining of the aorta in a normal rat (**a**,**e**), PCL (**b**,**f**), H-PCL (**c**,**g**), and VH-PCL groups (**d**,**h**). Arrowheads (**g**,**h**) indicate newly formed tissue layers. Scale bars = 1000 µm (**a**–**d**) and 400 µm (**e**–**h**); (**B**) histological analysis of vascular grafts stained with MT. The aorta in a normal rat (**a’**,**e’**), PCL (**b’**,**f’**), H-PCL (**c’**,**g’**), and VH-PCL groups (**d’**,**h’**) at 12 weeks post-implantation. Scale bars = 500 µm (**a’**–**d’**) and 100 µm (**e’**–**h’**); (**C**) the measurement of the vascular wall thickness from histology. The unit of the wall thickness is µm (the graph values shown are medians; *: Significance in *p* < 0.05 relative to Native). TEVG—tissue-engineered vascular graft; H&E—hematoxylin and eosin; PCL—poly(ε-caprolactone); H-PCL—heparin-AEMA-PCL; AEMA—2-aminoethyl methacrylate; VH-PCL—vascular endothelial growth factor (VEGF)-loaded heparin-AEMA-PCL; MT—Masson’s trichrome; L—lumen; I—tunica interna; M—tunica media; E—tunica adventitia; SMCs—smooth muscle cells.

Histological analysis of the H&E-stained normal rat aorta showed the tunica intima, which includes an endothelial layer; the tunica media, which includes elastic laminae with five to six layers of smooth muscle cells (SMCs); and the tunica adventitia (Figure 7A). In all experimental groups, the presence of a thin endothelial layer was observed on the luminal surface of the vascular grafts (Figure 7A). In addition, the H-PCL and VH-PCL groups showed an increase in cellularity in the vascular grafts, especially in the middle portion, which was lacking in the PCL group. In the H-PCL group, slight nanofiber degradation and newly formed microvessels were also found on the luminal surface of the vascular grafts, with spindle-shaped cells in the middle portion. In addition, giant cells were found along the outer layer of the vascular grafts, and macrophages were found inside the vascular grafts. There was more degradation on the luminal surface of the vascular grafts in the VH-PCL group than in the H-PCL group, with the presence of newly formed tissues, including spindle-shaped cells, at the degraded site. More spindle-shaped cells and microvessels were found arranged in a radial appearance in the middle portion of the vascular grafts in the VH-PCL group than in the H-PCL group. Although the peripheral surface of all vascular grafts showed a tunica adventitia–like layer, the thickest layer was found in the VH-PCL group. Intimal hyperplasia was observed in the H-PCL and VH-PCL groups, where the VH-PCL group showed thicker intimal hyperplasia than the former. MT staining showed the presence of collagen in all vascular grafts (Figure 7B). Collagen deposition on the luminal side and the adventitial layer was found in all experimental groups. However, the deposition of collagen was least in the PCL group, while it was dense and widespread on the luminal side and the adventitial layer in the VH-PCL group. Taken together, our histological and angiographical results showed that adequate wall thickness and favorable endothelialization in the H-PCL and VH-PCL vascular grafts, similar to those of the native aorta, occurred due to heparin and VEGF. The average wall thickness of normal rat aorta was 133.35 µm, whereas that of PCL, H-PCL, and VH-PCL grafts was 589.50, 627.22, and 667.84 µm, respectively (Figure 7C).

**Figure 8:**
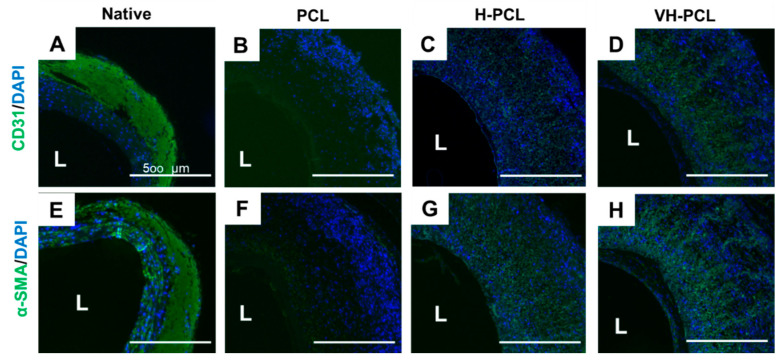
Representative immunohistochemistry images of a normal rat ((**A**,**E**) *n* = 3), PCL ((**B**,**F**) *n* = 4), H-PCL ((**C**,**G**) *n* = 4), and VH-PCL groups ((**D**,**H**) *n* = 4) at 12 weeks post-implantation in aged rats: (**A**–**D**) Green = CD31; blue = DAPI; (**E**–**H**) Green = α-SMA; blue = DAPI. L, lumen; PCL, poly(ε-caprolactone); H-PCL, heparin-AEMA-PCL; AEMA, 2-aminoethyl methacrylate; VH-PCL, vascular endothelial growth factor (VEGF)-loaded heparin-AEMA-PCL; DAPI, 4′,6-diamidino-2-phenylindole; α-SMA, alpha–smooth muscle actin.

Immunofluorescence staining showed the presence of CD31, a marker of the endothelial cells, as a monolayer on the inner surface of all vascular grafts (Figure 8A–D). In addition, α-SMA staining revealed remodeling of the grafted vessels, with the smallest number of α-SMA-positive cells observed in the PCL group. Generalized α-SMA staining was found in the H-PCL and VH-PCL groups, and radial-shaped alignments of α-SMA-positive cells were observed in the VH-PCL group (Figure 8E–H). However, several cell layers positive for α-SMA staining were also found in the VH-PCL group, indicating intimal hyperplasia (Figure 8H).

## 4. Discussion

Ideal small-diameter tissue-engineered vascular grafts (TEVGs) do not form blood clots inside the blood vessel and are suitable for treatment of high blood pressure, as well as being compatible with the native blood vessel structure. Several researchers have attempted to develop artificial vessel materials using biodegradable synthetic polymers, natural polymers, and decellularized xenografts [34]. PCL is a synthetic biodegradable polymer with a slow degradation rate, which leads to adequate tissue regeneration, excellent mechanical properties, and a patency rate that lasts for several months [25,35,36]. In this study, PCL with typical elasticity was manufactured using electrospinning to produce a 3D structure with sufficient porosity (~2 mm) for vessel support, a tensile strength of 0.52 MPa, and a burst pressure of 4560 mmHg. Stekelenburg et al. reported that in humans, the burst pressure of the saphenous vein is 1680−3900 mmHg, while that of the left internal mammary artery is 2000 mmHg [37]. Therefore, the vascular grafts developed in this study could be more suitable for small-diameter vascular grafting instead of for native human blood vessels.

The luminal surface of a TEVG is prone to thrombosis as it comes in direct contact with blood flow. In fact, synthetic small-diameter vascular grafts (<6 mm internal diameter) often experience blockage and fail due to thrombosis and intimal hyperplasia caused by decreased blood flow [38]. To overcome these issues, we developed heparin-immobilized electrospun PCL nanofibrous scaffolds using gamma irradiation. After heparin was chemically grafted onto the nanofiber surface via EDC/NHS chemistry, the amide and carboxyl groups of AEMA and heparin reacted with each other, while the bioactive components were preserved and exposed on the scaffold surface, which is favorable for cell attachment and proliferation later. We introduced VEGF into the heparin-immobilized PCL grafts to induce the movement, attachment, and proliferation of intravenous cells. VEGF was secured through its electrostatic binding with the negative-carriage characteristics of heparin.

When introducing additional heparin, the existing heparin conjugated onto the surface of biomaterials can cause high toxicity and non-uniform surface treatment by chemical agents, UV irradiation, and thermal discharge plasma [39,40]. To overcome these issues, gamma irradiation can be used to increase the grafting efficiency of biomaterial surfaces, as well as the uniformity of grafted bioactive molecules such as proteins and peptides, to increase hydrophilicity and cell viability [41,42]. Previously, we successfully developed PCL nanofibrous scaffolds using electrospinning, and grafted them with AEMA using gamma irradiation [17].

The surface properties of a polymer scaffold play a significant role in the cell–extracellular matrix (ECM) interaction. Of the many factors affecting the surface chemistry of a polymer scaffold, the hydrophilicity of the polymer surface is especially important for cell adhesion. Fewer cells attach to and proliferate on vascular grafts that have wettability of >90°. The water contact angle of 61° of the H-PCL vascular grafts in this study indicates that heparin and AEMA grafting successfully modify the surface wettability of PCL nanofibrous scaffolds.

Aging is strongly related to cardiovascular diseases, such as arteriosclerosis and varicose blood vessels, which can be treated using small-diameter vascular grafting [43]. Therefore, in animal studies, it is essential to conduct experiments on animals of a similar age as the target demographic in order to evaluate the exact efficacy of the developed vascular grafts.

Heparin has been used extensively for developing TEVGs for surface modifications to prevent thrombosis [1,44,45,46]. Janairo et al. reported that heparin-immobilized poly(l-lactide) (PLLA) vascular grafts show improved patency and increased cell infiltration compared to untreated grafts [1]. Therefore, heparin is believed to play an essential role in promoting cell infiltration into vascular grafts and has anti-thromboembolic effects. In addition, most cells that infiltrate into vascular grafts are vascular stem cells, progenitor cells, fibroblasts, and macrophages, and these cells are believed to promote TEVG remodeling and tissue regeneration [1]. VEGF is believed to facilitate colonization of the vascular endothelium and to increase cellularity in vascular grafts, similar to heparin [32]. Heparin has a high binding affinity with growth factors such as VEGF in the circulating blood, and can promote endothelial cell proliferation and migration [1]. Therefore, in this study, heparin immobilization and VEGF loading were successful in H-PCL and VH-PCL vascular grafts, which showed large numbers of infiltrated cells.

The middle part of vascular grafts, which most cells find difficult to infiltrate and consists of the tunica media in a blood vessel, is important for vascular tone regulation and ECM synthesis [47]. Overall α-SMA-positive cell infiltration was observed in the H-PCL and VH-PCL groups, with a few α-SMA-positive cells in the middle part of PCL grafts (Figure 8F–H). α-SMA is a marker protein involved in SMC contraction [48]. SMCs have two phenotypes—synthetic and contractile—and the phenotype of vascular SMCs is contractile [48]. This contractile phenotype enables the blood vessel to constrict or dilate, and is therefore important for vaso-activity [49]. In this study, the predominant cells in the middle part of the H-PCL and VH-PCL vascular grafts were SMCs with the contractile phenotype, which enabled these grafts to exhibit greater vaso-activity than the PCL vascular grafts.

In addition to the middle part of vascular grafts, luminal and adventitial surfaces are also important. The luminal surface comes in direct contact with blood flow, and incomplete endothelialization might lead to thrombosis [50]. In this study, heparin and VEGF together promoted the endothelialization of the VH-PCL vascular grafts. However, they can also affect the intimal hyperplasia in vascular grafts, especially VH-PCL vascular grafts. Several studies have investigated intimal hyperplasia in VEGF-coated vascular grafts, although the underlying mechanism is unclear [51,52]. Randone et al. suggested that VEGF results in higher SMC cellularity, which could induce altered stimuli generated from endothelial cells, such as higher basic fibroblast growth factor (bFGF) and lower transforming growth factor beta (TGF-β) [52]. These growth factors can stimulate SMC proliferation, which could cause intimal hyperplasia [52].

All vascular grafts showed foreign-body reactions in adventitial surfaces, especially in the H-PCL and VH-PCL groups. This reaction is normal and plays an important role in replacing polymer-based artificial blood vessels with native blood vessels [53].

The effects of aging on vessels vary. Aging can cause arterial changes, such as luminal dilation, intimal thickening, vascular stiffening, and endothelial dysfunction [54,55]. Aging can also change the migration of SMCs; aged SMCs migrate to the thickened intima, not the medial part of blood vessels [56]. As stem cells that can regenerate the cardiovascular system decrease with aging, it is more challenging to form a new middle layer of blood vessels [57]. In addition, aging can affect the vascular structure by initiating matrix promotion, which is essential for luminal dilation, intimal thickening, and vascular stiffening [54,55]. Aging also stimulates fibroblasts to increase collagen levels and cause intravascular matrix thickening [55].

Ungvari et al. studied changes in vascular endothelial cells and SMCs in aged rats [56]. To determine the difference in vessels between young and old rats, they used 6- and 24-month-old rats. Electron microscopy showed a significant decrease in the gross mitochondrial mass of the carotid arteries of old rats [57]. Moreover, measurements of cytochrome C oxidase (COX) activity and mitochondrial free-oxygen-radical (O_2_^•−^) and hydrogen peroxide (H_2_O_2_) production showed downregulation of COX expression and impaired mitochondrial biogenesis. Therefore, the decrease in mitochondrial mass might have caused the pathophysiological state of aged rats [56]. Increased mitochondrial oxidative stress might induce vascular inflammation in aged rats, and this stress is an important cause of cardiovascular dysfunction in the case of illness [58,59]. For these reasons, blood vessel transplantation in old rats cannot be considered a better option than that in young rats, and it is more likely that the blood vessels will worsen due to disease. However, our study showed remarkable results, such as favorable endothelialization and patency, high cellularity in the middle part of all vascular grafts, and a suitably formed adventitial surface, especially in the H-PCL and VH-PCL groups, compared to results obtained in young rats.

This study had a few limitations. We could not use many aged rats over 2 years old. The results might not be better than those of other studies because the tissue regeneration potential or systemic condition of aged rats is lower than that of young rats. Although several studies have suggested the need for TEVG assessment in old animals, no comparable evaluation has been performed to date [50,57]. In this study, we were able to evaluate our developed vascular grafts in aged rats to simulate an environment similar to that in older human beings, and there was a significant advantage in observing the effects of our vascular grafts in aged rats. In the future, we plan to perform experiments on young rats and compare the results with those obtained in this study.

## 5. Conclusions

We developed a modified method of increasing the functionality of PCL nanofibrous scaffolds using AEMA and heparin. This method can be applied in tissue engineering, especially for vascular grafts. The performance of the developed vascular grafts was analyzed using SEM, ATR-FTIR, TBO staining, and fibrinogen adsorption and surface wettability measurements, which showed that the electrospun PCL vascular grafts were uniformly modified by heparin and AEMA grafting, allowing endothelial cells to adhere well and spread with high cell viability on the vascular grafts. The H-PCL and VH-PCL vascular grafts showed excellent biocompatibility in the aged rat model. Therefore, we successfully achieved improvements in endothelial cell affinity and antithrombogenicity on a common biocompatible material using a practical modification method that is highly suitable for stimulating rapid endothelialization and effective antithrombosis.

## Data Availability

Not applicable.
